# Supplementary material for: Manganese doping for enhanced magnetic brightening and circular polarization control of dark excitons in paramagnetic layered hybrid metal-halide perovskites
Source: Nat Commun. 2021 Jun 9;12:3489. doi: 10.1038/s41467-021-23602-1 (PMC8190121; doi:10.1038/s41467-021-23602-1)
Supplement: Supplementary file 1 — Supplementary Information [file 41467_2021_23602_MOESM1_ESM.pdf]

SUPPORTING INFORMATION

Manganese doping for enhanced magnetic brightening and circular polarization control of dark excitons in paramagnetic layered hybrid metal-halide perovskites

Timo Neumann[1,2]*, Sascha Feldmann*[1]*, Philipp Moser*[2]*, Alex Delhomme*[3]*, Jonathan Zerhoch*[2]*, Tim van de Goor*[1]*, Shuli Wang*[4]*, Mateusz Dyksik*[4,5]*, Thomas Winkler*[1]*, Jonathan J. Finley*[2]*, Paulina Plochocka*[4,5]*, Martin S. Brandt*[2]*, Clément Faugeras*[3]*, Andreas V. Stier*[2]*, Felix Deschler*[2,*]

[1]Cavendish Laboratory, University of Cambridge, Cambridge, UK

[2]Walter Schottky Institut and Physik Department, Technische Universität München, Garching, Germany

[3]Université Grenoble Alpes, INSA Toulouse, Univ. Toulouse Paul Sabatier, EMFL, CNRS, LNCMI, Grenoble, France

[4]Laboratoire National des Champs Magnétiques Intenses, UPR 3228, CNRS-UGA-UPS-INSA, Grenoble and Toulouse, France

[5]Department of Experimental Physics, Faculty of Fundamental Problems of Technology, Wroclaw University of Science and Technology, Wroclaw, Poland

[*]corresponding author, Felix.Deschler@wsi.tum.de

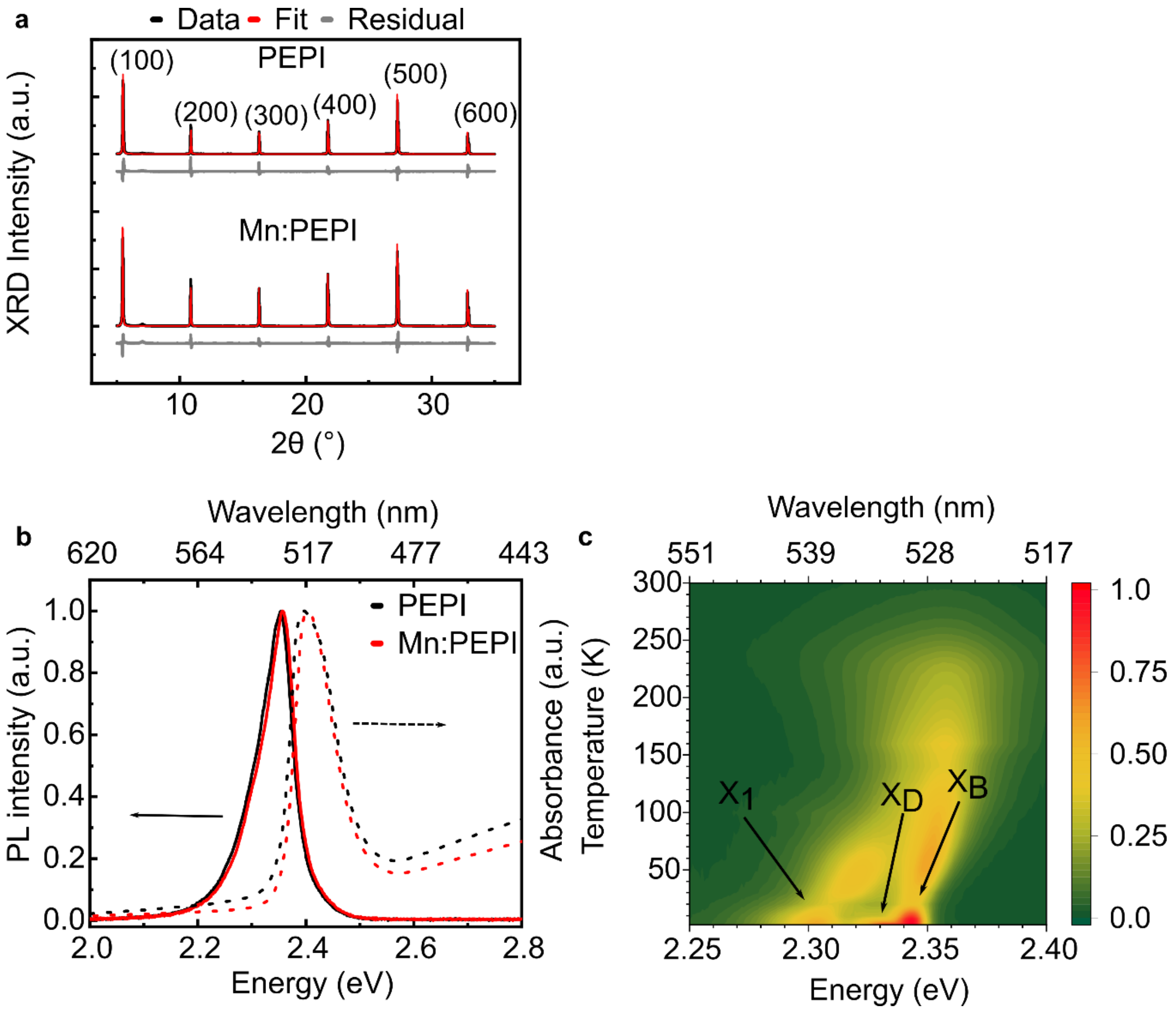

Figure S1: Structural and optical characterization of PEPI. a, XRD, **b**, UV/VIS absorption and PL (405 nm excitation) spectrum of PEPI and Mn:PEPI at room temperature. **b**, Temperature dependent PL of PEPI from 2 K to 300 K. X_1 denotes the broad emission (bound exciton), X_D the dark exciton and X_B the bright exciton.

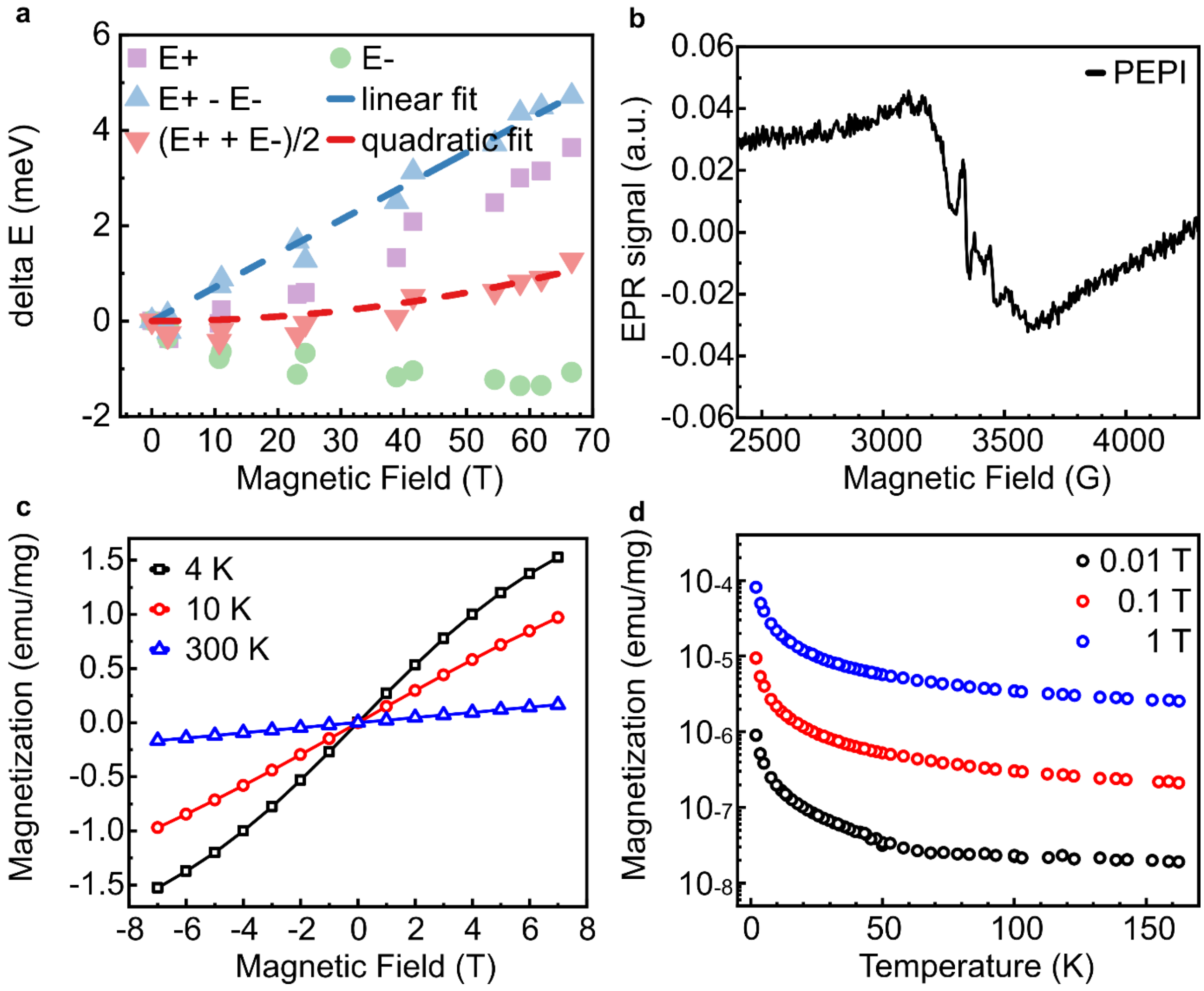

Figure S2: Optical and magnetic characterization of PEPI. a, Circularly polarized magneto-transmission of Mn:PEPI at 4 K. E+ and E- correspond to σ^+ and σ^- detection, respectively. Fits according to $\Delta E = \pm 1/2\, g\mu_B B + c_0 B^2$ yield g = 1.1, c_0 = 0.338 μeV/T^2. **b**, EPR signal of undoped PEPI perovskite. **c**, SQUID magnetization versus field sweep at different temperatures for undoped PEPI. **d**, Magnetization versus temperature for doped Mn:PEPI.

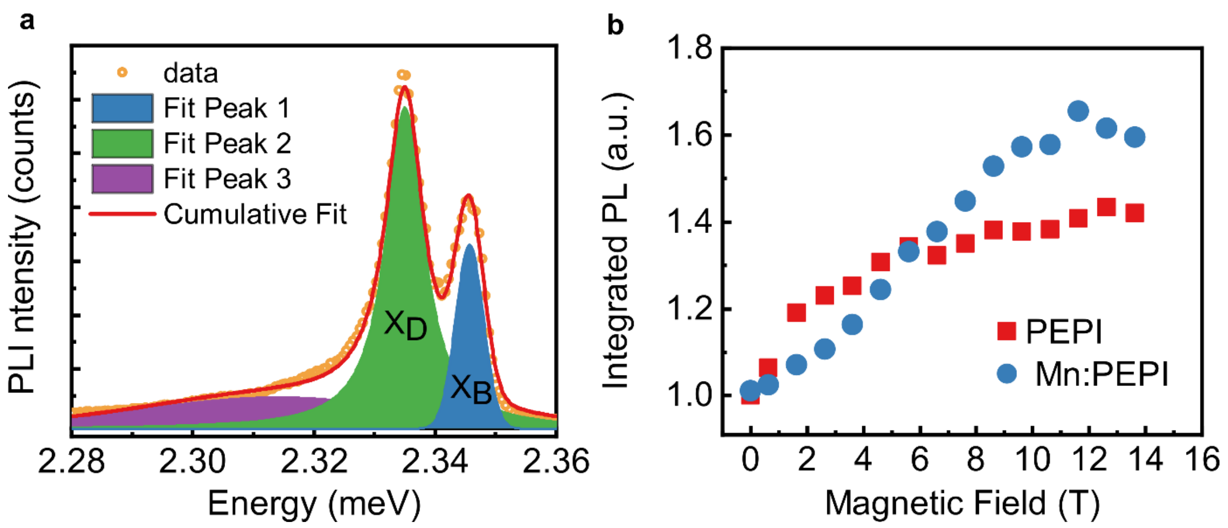

Figure S3: Magneto-Photoluminescence at 4 K. a, PL spectrum of PEPI at B = 14 T and 395 nm excitation, fitted with three Voigt profiles to assign the bright X_B and dark exciton X_D emission and the broad, low energy emission peak. Since relative intensities of bright and dark exciton differ among sample spots, we here chose a spectrum with well-distinguishable peaks, while in the main text a spectrum with maximum brightening and CPL. **b**, relative change in integrated (2.2 eV to 2.4 eV) PL intensity. The overall PL emission increases up to 40% for PEPI and 60% for Mn:PEPI.

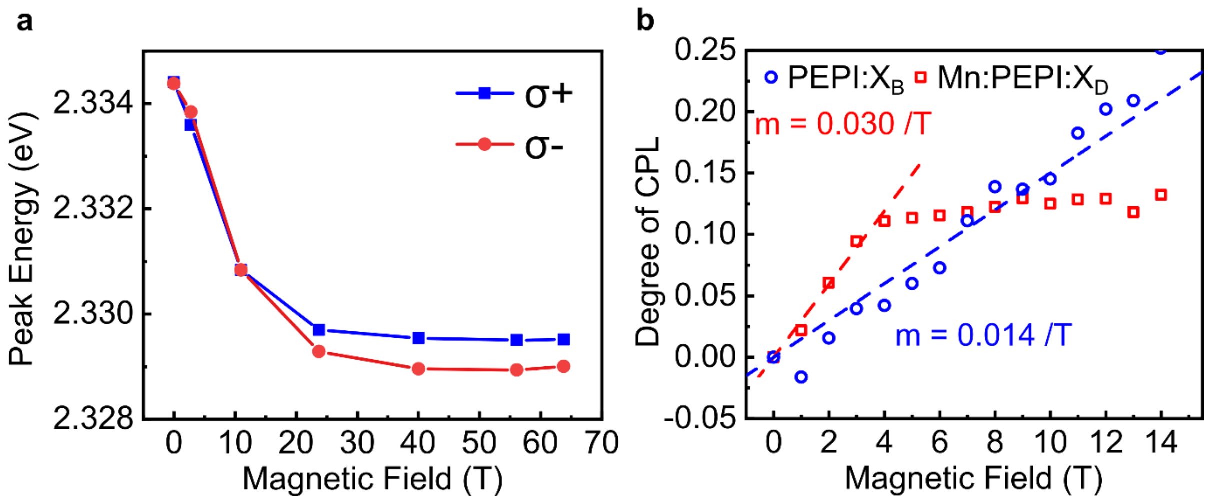

Figure S4: PL energy and polarization at 4 K. a, PL peak energy for both circular polarizations of Mn:PEPI: X_D with a ~100ms pulsed magnetic field in Faraday geometry and excitation at 405 nm. A small Zeeman shift of the dark exciton emission only becomes measurable for B > 20 T. **b**, Circular polarization of Mn:PEPI: X_D and PEPI: X_B with linear fit. Upon Mn doping, the previously unpolarized X_D peak shows CPL with a twofold steeper increase than the bright exciton emission of the undoped sample.

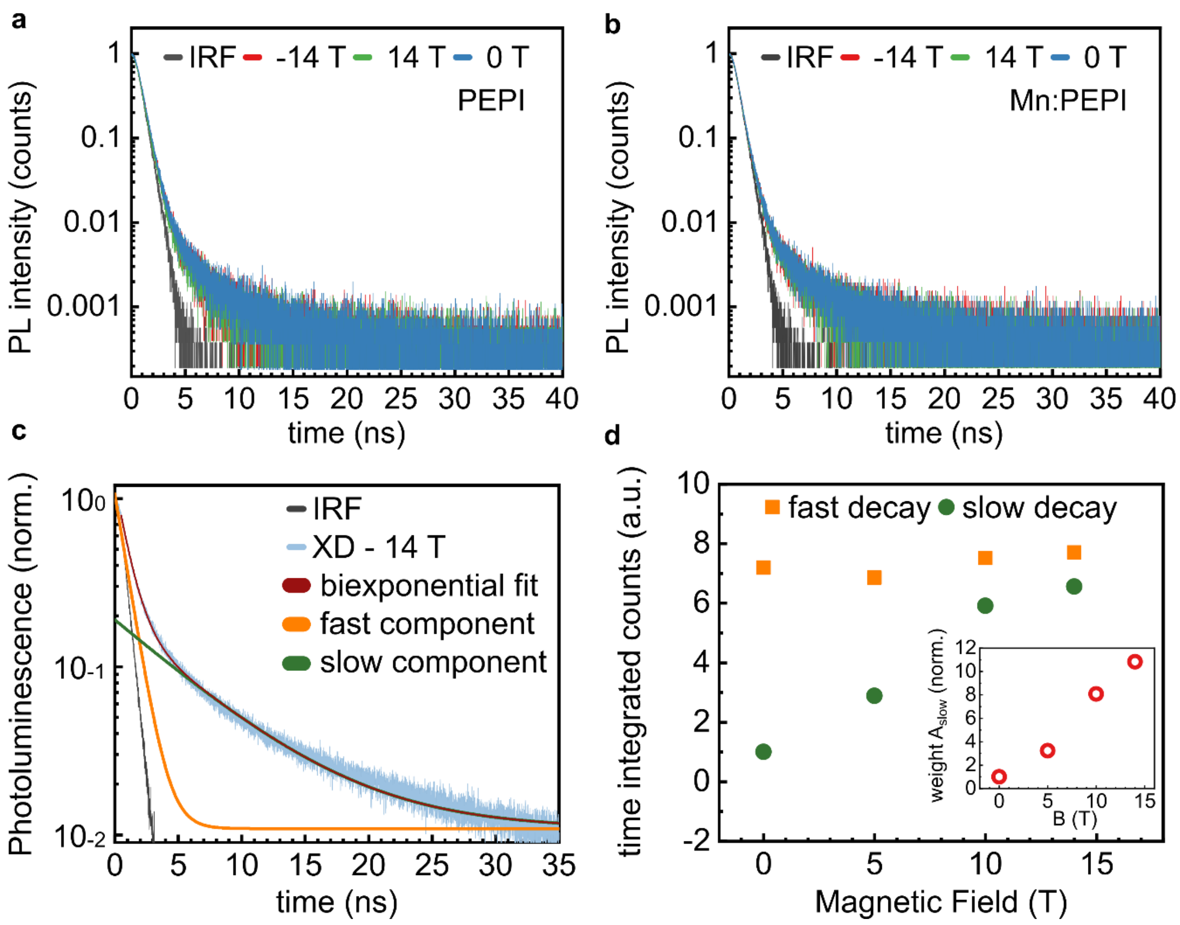

Figure S5: Transient circularly polarized PL at 4 K of PEPI films. a, bright exciton in PEPI, **b**, bright exciton in Mn:PEPI. The bright exciton PL kinetics remain unaffected by magnetic field strength, direction, and magnetic doping. **c**, dark exciton biexponential fit and its two components. **d**, time integrated PL for the fast and slow component as a function of magnetic field. While the fast component intensity remains unaffected by the magnetic field, the slow component increases ~sixfold (due to higher weight A_{slow},inset), which identifies the slow component as the dark exciton emission.

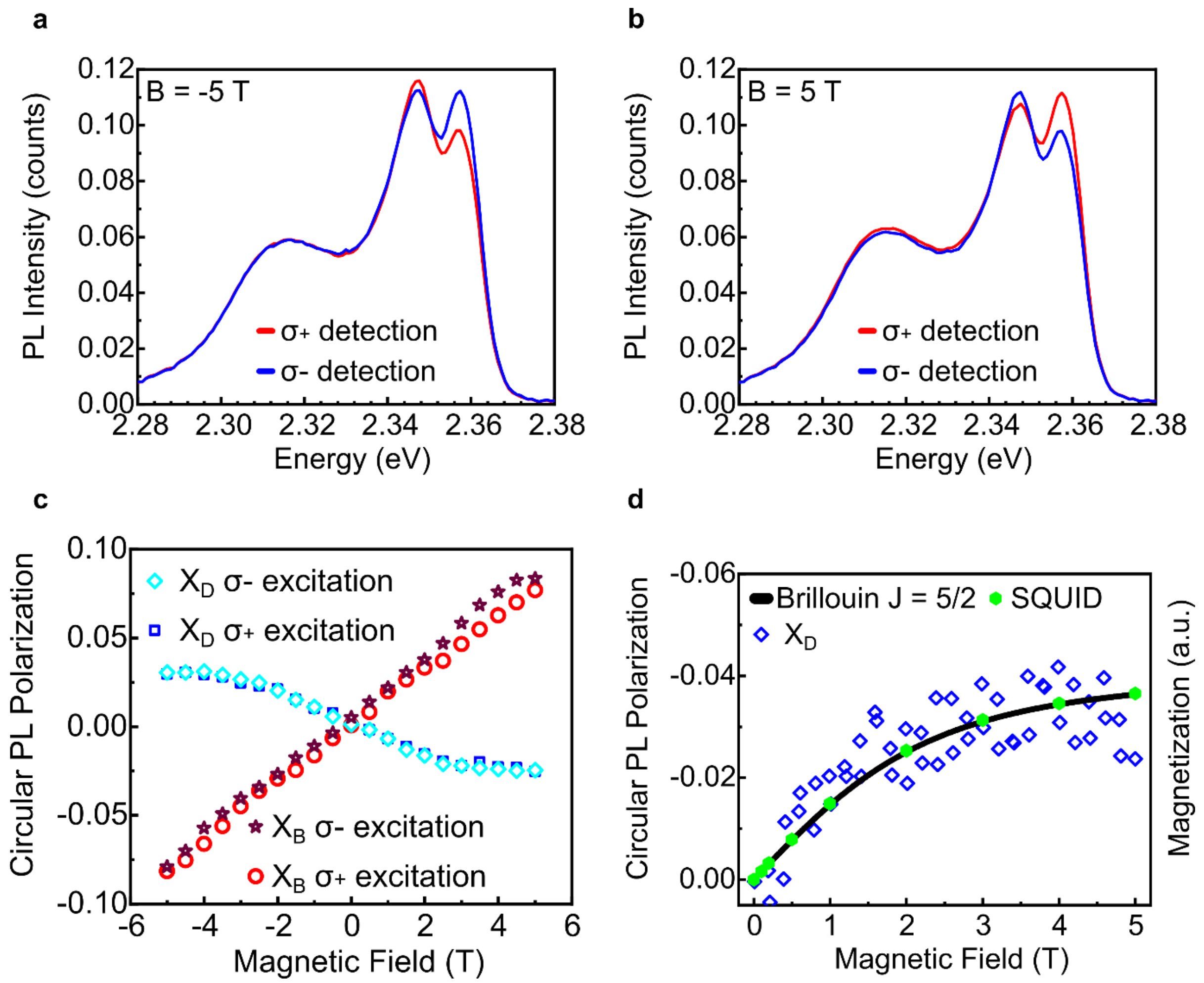

Figure S6: Low temperature (4 K) circularly polarized magneto-PL of Mn:PEPI. a & b, PL spectra for positive and negative magnetic field in Faraday geometry with σ- excitation at 405 nm. **c**, Degree of circular polarization for dark and bright exciton for both excitation polarizations. **d**, Proportionality of CPL polarization and magnetisation. The sample comes from a different batch than that in the main text and saturates at a lower polarization. The measurements with co-/ and counter-polarization between excitation and detection confirm the circular nature of the polarization and show that the polarization of the excitation has no impact on the polarization of the emission.

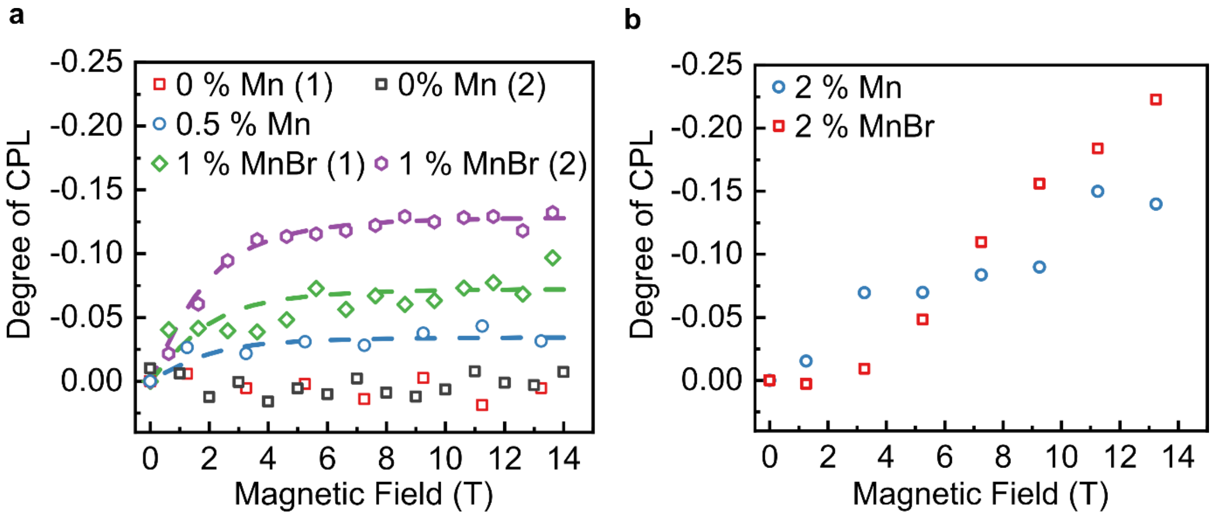

Figure S7: Effect of precursor salt, manganese concentration and sample spot on degree of circular polarization . a, Mn concentration 0%, 0.5%, 2% at different sample spots (1) and (2). **b**, 2% Mn. Circular polarization occurs for different precursors and concentrations but shows local fluctuations. At higher concentrations, a different behaviour occurs possibly due to Mn-Mn interactions.

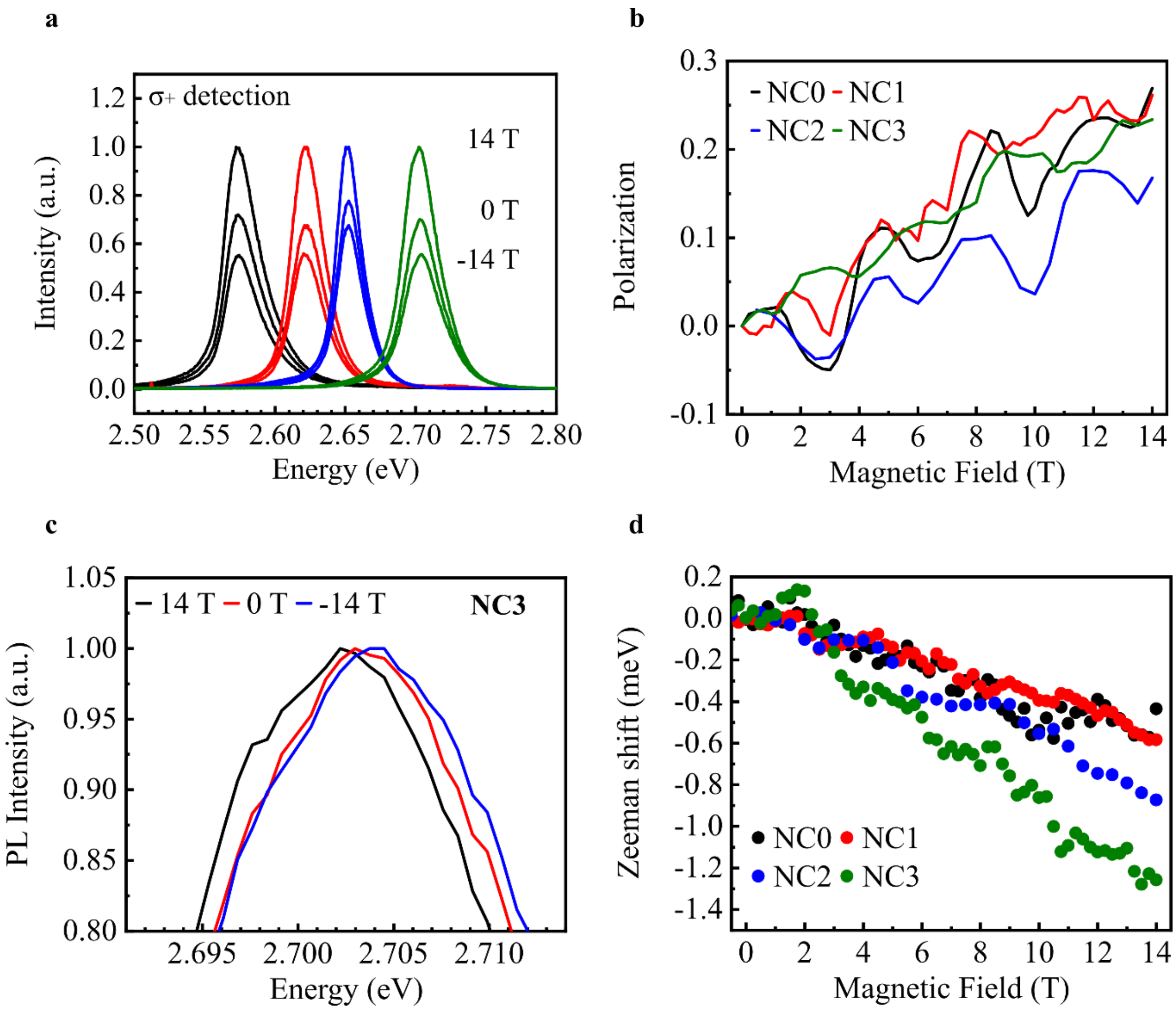

Figure S8: Low temperature (4 K) circularly polarized magneto-PL of manganese doped $CsPbBr_3$ nanocrystals. a, PL spectra (405 nm excitation) of NCs with 0%, 0.1%, 0.2%, 0.3% Mn content (from black to green). **b**, Circular PL polarization. **c**, Normalized PL spectra for NC3. **d**, Zeeman shift of peak position with magnetic field. In these perovskite nanocrystals Mn doping has no impact on the magneto-PL, contrary to our findings for Mn:PEPI.
